# Understanding kitten fostering and socialisation practices using mixed methods

**DOI:** 10.1017/awf.2024.45

**Published:** 2024-11-11

**Authors:** Courtney Graham, Katherine E Koralesky, David L Pearl, Lee Niel

**Affiliations:** 1 Department of Clinical Studies, Ontario Veterinary College, University of Guelph, Guelph, ON, Canada; 2 Department of Population Medicine, Ontario Veterinary College, University of Guelph, Guelph, ON, Canada; 3 The Campbell Centre for the Study of Animal Welfare, University of Guelph, Guelph, ON, Canada; 4 Animal Welfare Program, Faculty of Land and Food Systems, University of British Columbia, Vancouver, BC, Canada

**Keywords:** adoption, animal welfare, cat, qualitative, survey, volunteer

## Abstract

Many companion kittens entering shelters are fostered by volunteer community members during the sensitive period for socialisation (~2 to 9 weeks of age) when early experiences are critical to behavioural development. Using a mixed-method survey, we explored current fostering practices relevant to kitten behavioural development and welfare. Foster caretaker participants (n = 487) described their fostering practices and reported providing kittens with a majority of recommended socialisation experiences, such as handling and exposure to various toys and exploratory items. In open-ended text responses, foster caretakers described how they adapted socialisation practices for fearful kittens and the supports and challenges they perceived to impact their ability to properly socialise kittens. Some non-recommended techniques (e.g. flooding) were reported for socialising fearful kittens, with a decreased odds of reporting non-recommended techniques for participants with a higher level of agreeableness personality trait and an increased odds of reporting if fostering practices had been impacted by the COVID-19 pandemic. Foster caretakers reported feeling supported through shelter-supplied resources, personal knowledge, external support, and having access to socialisation opportunities; however, faced personal (e.g. time constraints), shelter-specific (e.g. lack of shelter support), and kitten-specific challenges (e.g. kitten illness). This study highlights the perspectives of foster caretakers as related to optimal socialisation, behavioural development, and welfare. To identify opportunities for improvement it is important to investigate the socialisation guidelines provided to foster caretakers, with the ultimate goal of enhancing kitten behavioural development for improved welfare, long-term adoption, and caretaker satisfaction.

## Introduction

Prior to adoption, many companion kittens entering shelter systems spend the early weeks of their lives in foster care with volunteer community members, primarily due to their vulnerable health status and need for socialisation (Dinnage *et al.* 2009; Attard *et al.* 2013; The Association of Shelter Veterinarians 2022). This time in foster care generally overlaps with the sensitive period for socialisation, which in kittens is from approximately 2 to 9 weeks of age (Karsh 1984; Karsh & Turner 1988; Lowe & Bradshaw 2001; Bateson 2014). Therefore, kitten foster caretakers hold a unique and important role in the behavioural development of kittens within their prime socialisation period.

Expert recommendations for early kitten rearing typically include suggestions for activities involving exposure to physical and social situations and contexts. These activities can include positive social interactions with people and other animals, play engagement, exposure to common household stimuli, and access to vertical space, safe hiding areas, and scratching materials (American Association of Feline Practitioners 2004; Seksel 2008). Shelter-provided guidelines for kitten rearing in Canada and the USA include similar recommendations with particular focus on fostering practices (e.g. BC SCPA undated; San Franscisco SPCA undated).

Experiences during the socialisation period impact long-term behaviour and welfare of kittens. Cats provided with additional socialisation opportunities as kittens, such as handling by multiple people and exposure to common household sounds, showed less fearful behaviour towards humans, and caretakers reported being strongly attached to their cats when assessed at one year of age (Karsh 1984; McCune 1995; Casey & Bradshaw 2008). For example, kittens handled for 40 min a day between 3 and 7 weeks of age spent more time in close contact with people than kittens who received handling for 15 min a day when tested at one year of age (Karsh 1984). Further, cats who were handled as kittens within their socialisation period spent more time in proximity to people as well as showing decreased latency to approach people when tested a year later compared to cats who had not been handled as kittens aside from regular cleaning and feeding routines (McCune 1995; Casey & Bradshaw 2008). In contrast, situations that provoke fear in kittens can potentially lead to stress-related health issues (e.g. respiratory illness; Bannasch & Foley 2005), as well as behavioural concerns such as aggression or inappropriate elimination (Levine 2008). Such issues can impact animal welfare, negatively influence the human-animal bond, and lead to mistreatment, relinquishment to shelters, and/or ultimately to euthanasia (DiGiacomo *et al.* 1998; Salman *et al.* 1998, 2000; Coe *et al.* 2014).

Research investigating kittens or cats in foster homes or shelters has primarily been focused on outcomes of health (e.g. Strong *et al.* 2020) or length of stay (e.g. Janke *et al.* 2017), and few have focused on behavioural outcomes related to kitten rearing beyond basic socialisation research as described above. Research has investigated the relationship between human and caretaker personality traits and cat behaviour (e.g. higher caretaker openness was associated with cats who were less anxious; Kotrschal *et al.* 2014) and dog behaviour (e.g. higher caretaker extraversion was associated with lower caretaker-reported dog aggression; Kuroshima *et al.* 2016). Other studies investigating the motivations of volunteer assistance puppy raisers found that intrapersonal factors, social support, and puppy characteristics were important aspects for puppy raisers’ perceived ability to raise puppies effectively (Mai *et al.* 2020). Factors that hindered puppy raisers’ motivations included other commitments and puppy behaviour (e.g. fearful avoidance, high energy levels), whereas factors that facilitated motivations included having a network of additional supports and access to preferred learning modalities (Mai *et al.* 2021). Similar studies have not been conducted with kittens or cats or in the foster context.

While some research has been conducted to broadly investigate animal volunteer attitudes and perceptions (e.g. Reese *et al.* 2021), to date there have been no studies specifically focused on kittens or foster caretaker experiences. Given the important role foster caretakers play as regards the behavioural development of kittens, it is important to understand how their experiences, strategies, and characteristics influence kitten-rearing practices. Thus, the overall goal of the current study was to use a mixed-method survey to understand fostering experiences, practices used to socialise kittens, approaches to mitigating fear, and the resources foster caretakers use and need to improve kitten welfare. Applying a mixed-method approach (i.e. with both quantitative and qualitative data components) improves the rigour of a study by collecting data in multiple forms to thereby provide a richer viewpoint of the questions being explored than either approach alone (Carter *et al.* 2014; Creswell & Creswell 2018; Miles *et al.* 2020). In companion animal research, mixed methods have been used to understand caretaker motivations for dog acquisition (Packer *et al.* 2020; Holland *et al.* 2022), investigate communication trends among veterinarians and companion animal caretakers (Janke *et al.* 2022), and explore perspectives around impacts of the COVID-19 pandemic on veterinary care (Muzzatti & Grieve 2022; Owczarczak-Garstecka *et al.* 2022) and companion animal caretaker well-being (Clements *et al.* 2021). Findings from the current survey may be used to improve socialisation strategies, early life management, and behavioural development practices, thus improving the welfare of companion kittens and increasing the likelihood that they will be retained in their adoptive homes.

Our first objective was to explore the types and timing of socialisation opportunities provided to kittens, as well as foster caretaker perspectives on kitten behaviours and characteristics perceived as important for successful and lasting adoption. The second was to use qualitative methods to explore: (1) how kitten foster caretakers adapt approaches to socialising a kitten perceived as fearful; (2) which resources they feel are most important for providing optimal socialisation; and (3) which challenges they face that reduce their ability to conduct optimal socialisation. Our third and final objective was to use quantitative methods to investigate factors associated with use of recommended socialisation practices. We used a cross-sectional design and did not initially employ any overt hypotheses, apart from our prediction that foster caretakers with more experience (in terms of years fostering and numbers of litters fostered) would have greater understanding of optimal fostering practices for kittens.

## Materials and methods

### Survey design and reflexivity statement

Our online survey (Table S1; Supplementary material) was hosted through Qualtrics® survey software (Qualtrics Software Company, Provo, UT, USA) and was available worldwide from 21 July to 20 August 2021. Survey participation was open to foster caretakers of young kittens (8 weeks of age or younger) who were at least 18 years of age and who had fostered kittens within the two years prior to participation. Internet access was required to participate with recruitment occurring via convenience sampling with a snowball approach on social media platforms (Facebook, Twitter/X, TikTok) on personal and institutional webpages (OVC Companion Animal Behaviour and Welfare Lab). Prospective participants could respond to a social media advert and share on their personal platforms. Additionally, humane societies and charities, professional cat bloggers, and other related shelter and animal behaviour contacts were emailed the advertisement (Figure S1; Supplementary material) and survey description and asked to share with their communities. The survey protocol was reviewed and approved by the University of Guelph Research Ethics Board and conformed to all federal and provincial guidelines governing the use of human participants in research (REB #21-05-007).

The survey consisted of multiple- and single-choice questions, Likert scales, and open-ended questions. It included questions on foster caretaker demographics (including personality traits using the validated Ten-Item Personality Index [TIPI]; Gosling *et al.* 2003) and previous experience with kittens, socialisation practices and techniques used with kittens under 8 weeks of age, general perceptions of kitten behaviours in terms of enhancing or reducing the success of adoption, and resources needed and challenges faced by foster caretakers to provide optimal socialisation (Table S1; Supplementary material).

Reflexivity statements provide an opportunity for researchers to share their values, beliefs, and personal background. Sharing this information acknowledges that these aspects shape the research approach and data interpretation, and can thus add credibility to a study (Creswell & Miller 2000; Holmes 2020). CG and KK conducted the qualitative analyses. CG is primarily experienced in quantitative methods to address questions of companion animal welfare and behaviour using an affective states framework (Duncan 1993) and has incorporated qualitative methods into her research to expand her approach. Her work incorporates the perspectives of animal caretakers to gain a better understanding of how to improve the welfare of the animals being cared for. KK uses a variety of qualitative research methods, is a long-standing animal shelter volunteer, and has previous experience fostering cats. She applies an understanding of the human factors that influence animal welfare to improve human and animal welfare outcomes.

### Qualitative analysis

Open-ended responses were analysed using thematic analyses following an inductive (i.e. data driven) descriptive approach (Miles *et al.* 2020; Saldaña 2021). Responses were read in full by CG, who then created codes (i.e. short phrases or words) that described participant responses to each question. Entire responses, or sections of responses, could be coded under multiple codes. Using NVivo qualitative data analysis software (V1.6.1, QSR International, Burlington, MA, USA), CG developed a codebook (i.e. a document that defines each code, provides examples of the code, and describes when to use different codes) for each open-ended question (MacQueen *et al.* 1998). To assess reliability, KK used the codebook to code 15% of responses in random order and coding agreement was investigated within NVivo (MacQueen *et al.* 1998). Codebooks were uploaded to compare analyses and any discrepancies were discussed in detail to ensure consensus. Following initial coding and coding reliability, codes were categorised to construct themes and sub-themes based on relational patterns of responses (Saldaña 2021). Additionally, as done in other studies with large numbers of open-ended responses (e.g. Packer *et al.* 2020), code frequencies were measured to provide an insight into their prominence within each theme (Creswell & Plano-Clark 2007; Sandelowski *et al.* 2009). Each qualitative code was given a binary quantitative value of being mentioned or not for each participant and was included in the quantitative dataset and statistical model.

### Quantitative analysis

Following initial cleaning of data in Microsoft® Excel for Mac (v16.58), statistical analyses were conducted in Stata statistical software (v15.1 for Mac, StataCorp 2015, College Station, TX, USA). Through the qualitative analyses, we identified a number of participants who reported using techniques that are not currently recommended when providing socialisation to a fearful kitten, such as ignoring the kitten, not providing hiding spots, limiting agency, or flooding the fearful kitten with the exposure, which involves forcing the interaction at full intensity (Yin 2009; Landsberg *et al.* 2013; Špinka & Wemelsfelder 2018; Špinka 2019; Riemer *et al.* 2021). Our main quantitative outcome was therefore whether or not foster caretakers mentioned the use of a technique that is not recommended when socialising a fearful kitten. We fitted a multivariable logistic regression model to identify factors associated with this outcome, including foster caretaker demographics, personality traits, previous experience with kittens or cats, socialisation opportunities provided, use/provision of shelter guidelines, as well as mention of other qualitative codes (see codes in *Qualitative results*).

Prior to fitting our model, we assessed whether variables were highly correlated to avoid issues concerning collinearity using Spearman rank, Phi, and Pearson correlation coefficients. If two variables were highly correlated (i.e. >|0.70|), the most meaningful variable for the outcome was selected for further analysis. The assumption of linearity between continuous independent variables and the outcome on a log odds scale was graphically assessed using locally weighted regression curves (LOWESS) and by testing the inclusion of a quadratic term in the model. If the relationship was non-linear and could not be appropriately modeled as a quadratic relationship, the continuous variable was categorised. Initially, we fitted univariable regression models and considered including variables for multivariable analysis if they were significant using a liberal significance level (α = 0.20). All variables significant in the univariable analyses were included in a main effects model and were removed in a manual backward stepwise fashion. Variables were retained in the multivariable model if they were statistically significant (α = 0.05), part of a statistically significant interaction, or were considered an explanatory antecedent or distorter variable (i.e. a confounder). Confounding variables were non-intervening variables that caused a change of greater than 20% in the coefficient of other statistically significant variables in the model when removed (Dohoo *et al.* 2014). Two-way interactions were evaluated among all variables initially considered for inclusion in the multivariable model. The final model fit was assessed using a Pearson goodness-of-fit test or a Hosmer-Lemeshow goodness-of-fit test, depending on whether the data were binomial or binary, respectively. Pearson and deviance residuals were assessed to identify any potential outliers. Other diagnostics (i.e. leverage, influence) were also assessed to evaluate impacts on the model.

## Results

### Survey responses

In total, 683 responses were received. Nineteen responses were removed for not providing appropriate consent, 57 responses were removed for answering zero questions, and 120 responses were removed for not answering a sufficient proportion of questions (> 70% — to ensure participants had answered a majority of questions while also retaining as many responses as possible), leaving 487 responses for the final analyses.

### Descriptives

The majority of foster caretaker respondents resided in mid-sized communities (193/483; 40.0%) in the United States (334/482; 69.3%), identified as women (450/485; 92.8%), and had worked or volunteered with animal shelters (218/487; 44.8%) (Table 1). On average, participating foster caretakers were 43.5 years of age (range: 18 to 86 years), had fostered 5.1 litters of kittens annually (range: 1 to 20+) and 11.9 litters over their lifetime (range 1 to 20+), for 5.7 years (range: 0 to 20+ years) through an average of two shelters (range: 0 to 20+). Average scores for personality traits for agreeableness, conscientiousness, and emotional stability were above the population average (Table 1), whereas extraversion was below the population average, and openness to experience was equivalent to the population average (Gosling *et al.* 2003).

**Table 1.** Demographic characteristics of kitten foster caretaker survey participants, based on survey question response

Characteristic	N (%)
Sex (n = 485)	
Woman	450 (92.8)
Man	20 (4.1)
Non–binary	8 (1.7)
Prefer not to answer	7 (1.4)
Country of residence (n = 482)	
United States	334 (69.3)
Canada	92 (19.1)
Australia	27 (5.6)
Other^a^	29 (6.0)
Community type (n = 483)	
Rural (fewer than 4,999 people)	49 (10.1)
Small (5,000 to 49,999 people)	78 (16.2)
Mid–sized (50,000 to 999,999 people)	193 (40.0)
Large (more than 1 million people)	163 (33.8)
Advanced knowledge^b^ (n = 487)	
Cat or dog trainer/Behaviour consultant	29 (6.0)
Veterinarian/Veterinary assistant	88 (18.1)
Shelter worker/volunteer	218 (44.8)
Animal behaviour/welfare researcher	36 (7.4)
Other position	33 (6.8)
Socialisation practices impacted by the pandemic (n = 486)	
No, there have been no changes	232 (47.7)
Only fostered during the pandemic	75 (15.4)
Yes, the pandemic impacted practices	179 (36.4)
Personality trait averages (n = 485)^c^	Average [range; SD]
Extraversion	3.90 [1, 7; SD = 1.61]
Agreeableness	5.53 [2.5, 7; SD = 1.04]
Conscientiousness	5.83 [1.5, 7; SD = 1.13]
Stability	4.96 [1, 7; SD = 1.33]
Openness to experience	5.35 [2, 7; SD = 1.07]

a Other countries included: Cyprus (n = 1), Finland (n = 4), France (n = 1), Germany (n = 2), Jordan (n = 1), Mexico (n = 1), New Zealand (n = 3), Portugal (n = 1), South Africa (n = 1), Spain (n = 4), Sri Lanka (n = 1), United Arab Emirates (n = 1), and United Kingdom of Great Britain and Northern Ireland (n = 8).

b Veterinary positions also included veterinary technician, nurse, student, or other position within a veterinary clinic setting; shelter positions also included other positions within a shelter or rescue setting; animal behaviour/welfare researcher positions also included student, human-animal interaction researcher, or professor positions. “Other” positions included groomer, breeder, farm manager, pet sitter, SPCA animal inspector, dog boarding manager, zookeeper, among others.

c Population averages (Gosling *et al.* 2003): Extraversion: 4.44 (SD = 1.45), Agreeableness: 5.23 (SD = 1.11), Conscientiousness: 5.40 (SD = 1.32), Emotional stability: 4.83 (SD = 1.42), Openness to experience: 5.38 (SD = 1.07)

The majority of respondents had other cats (409/487; 84.0%) or dogs (260/487; 53.4%) in the home, provided controlled and supervised interaction between foster kittens and household pets (279/487; 57.3%), spent more than 3 h daily interacting with foster kittens (227/487; 46.6%), and gave names to their foster kittens (382/487; 78.4%).

### Socialisation opportunities provided

**Figure 1.**
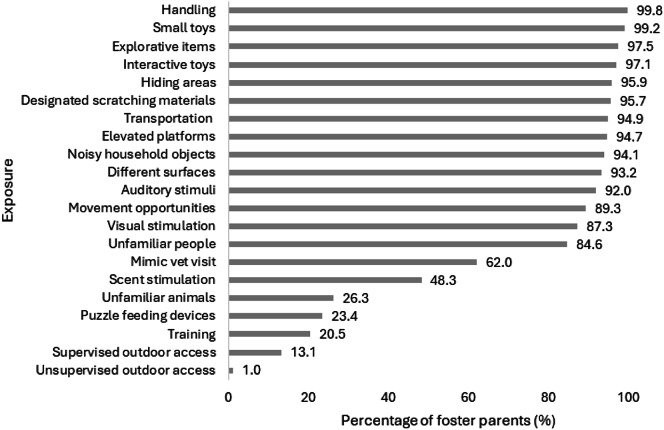
The percentage of foster caretakers (n = 487) providing exposures for socialisation opportunities to kittens.

Most foster caretakers provided access to the different socialisation opportunities examined (Figure 1), with the highest proportion providing exposure to handling (486/487; 99.8%), small toys (483/487; 99.2%), explorative items (475/487; 97.5%), and interactive toys (473/487; 97.2%), and the lowest proportion providing exposure to unfamiliar animals (128/487; 26.3%), puzzle feeding devices (114/487; 23.4%), training (100/487; 20.5%), and supervised (64/487; 13.1%) and unsupervised outdoor access (5/487; 1.0%).

**Figure 2.**
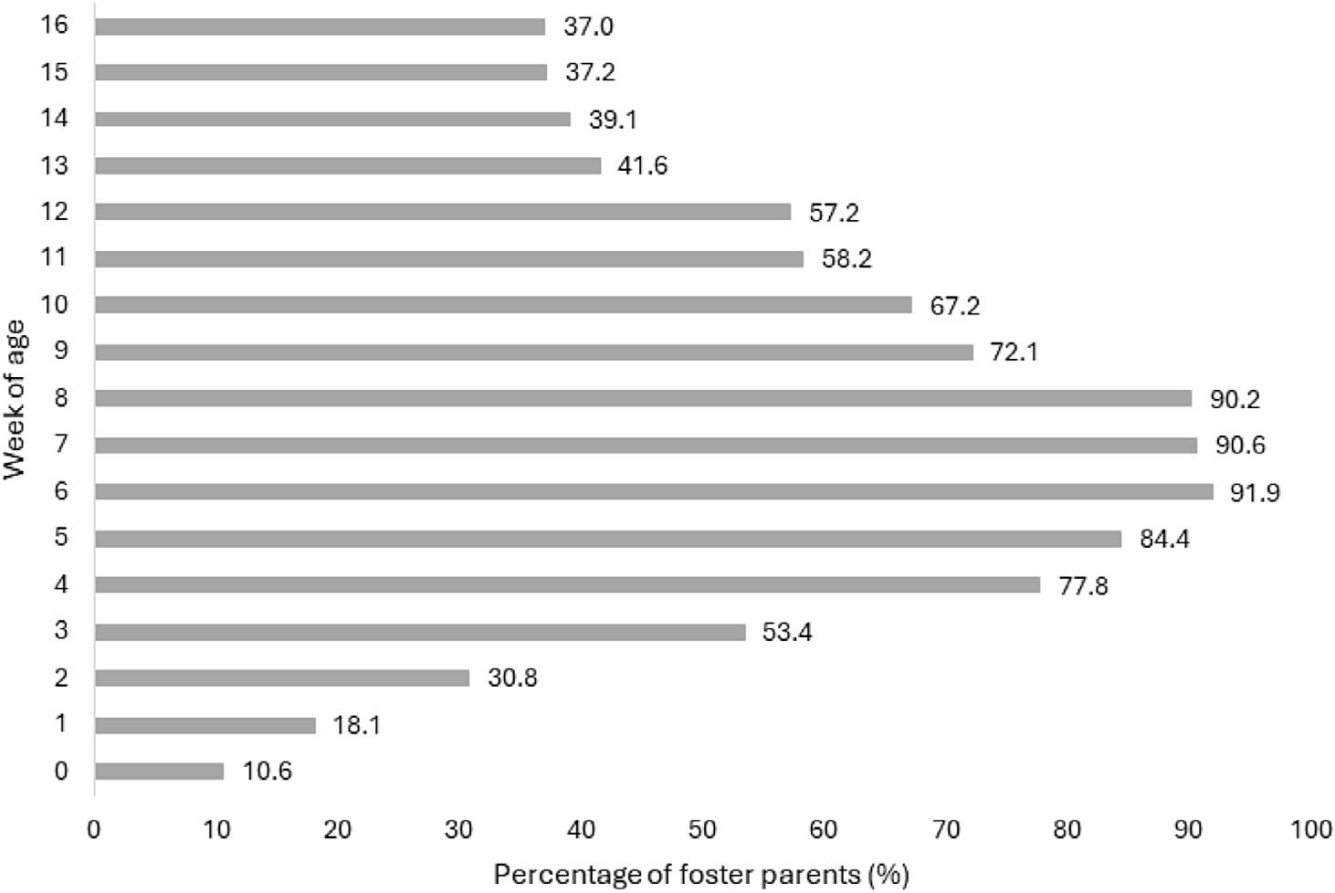
Kitten age (weeks) that foster caretakers (n = 481) focused on for providing socialisation experiences with the option to select all weeks that apply.

Most foster caretakers focused on weeks 6 (442/481; 91.9%), 7 (436/481; 90.6%), and 8 (434/481; 90.2%) of age for providing socialisation experiences to their kittens. Within the sensitive period for socialisation, foster caretakers focused the least on providing socialisation experiences during weeks 2 (148/481; 30.8%) and 3 (257/481; 53.4%) (Figure 2). The average estimated age of adoption was 9.9 weeks (range: 5 to 20+ weeks).

### Kitten characteristics for adoption success

**Figure 3.**
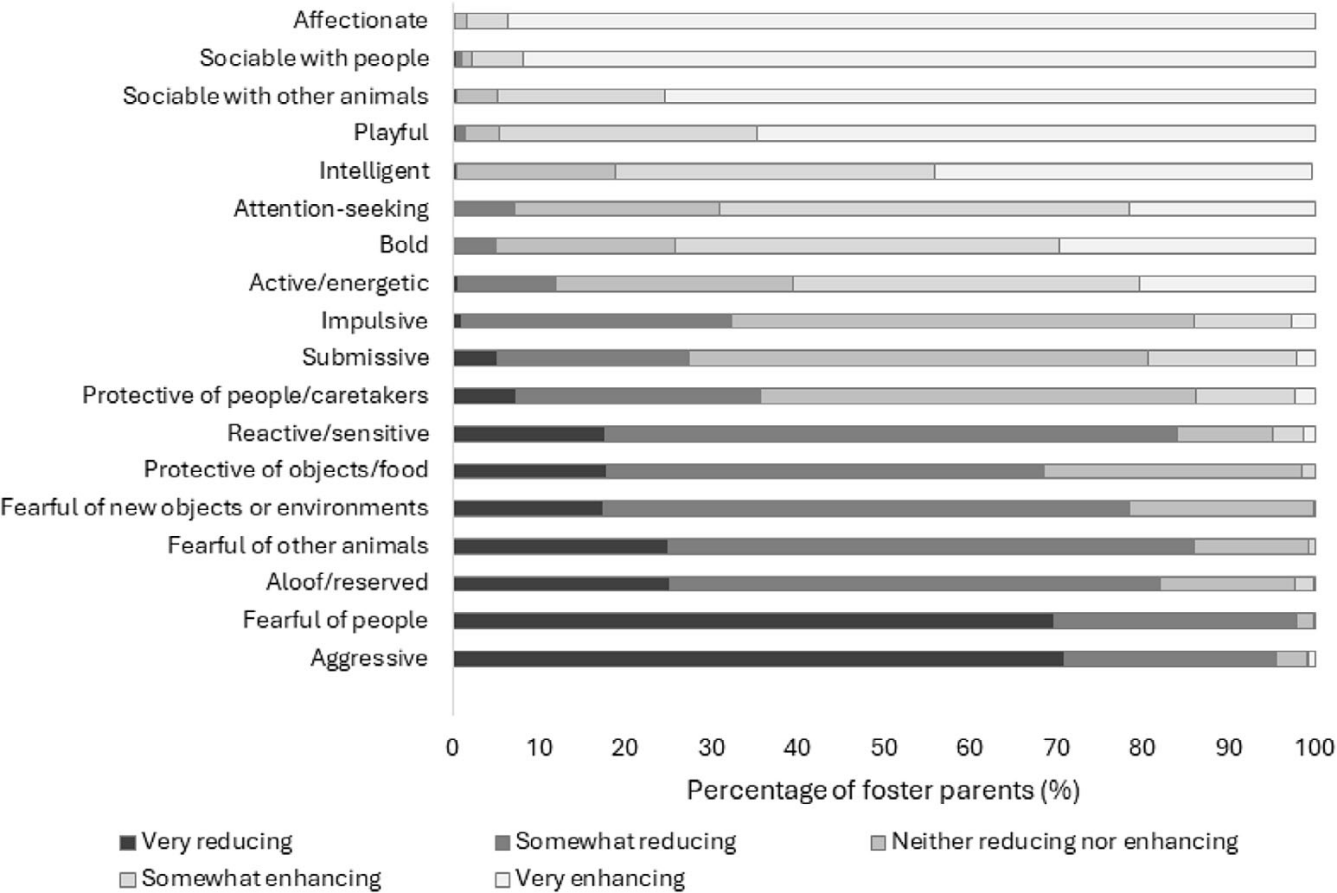
Kitten behaviours or characteristics that were perceived by foster caretakers (n = 487) to either enhance or reduce a kitten’s likelihood of being successfully adopted and retained in the adoptive home.

In regard to kitten characteristics that were perceived as enhancing or reducing a kitten’s likelihood of being successfully adopted and retained in the adoptive home, foster caretakers selected ‘affectionate’, ‘sociable with people’, ‘sociable with other animals’, and ‘playful’ as the most enhancing kitten characteristics, and ‘aggressive’, ‘fearful of people’, ‘aloof/reserved’, and ‘fearful of other animals’ as the most reducing kitten characteristics (Figure 3).

### Qualitative results

We describe themes and sub-themes for three open-ended questions: (1) how foster caretaker participants socialise a fearful kitten; (2) resources needed to conduct socialisation; and (3) barriers or challenges to conducting socialisation. We include direct quotes to exemplify sub-themes (randomly generated participant IDs follow each quote). The proportion of responses reported are calculated from the number of participants who provided an answer to each question.

### Socialising a fearful kitten

Participants described a variety of approaches to providing socialisation to a kitten who is reacting fearfully to a new exposure which we categorised into two main themes: (1) the process of providing socialisation; and (2) how they considered individual kitten traits when providing socialisation. Of the 487 responses, 440 participants answered this question.

The ‘process of providing socialisation’ included ‘going slowly’ (n = 258; 58.6% of responses), providing ‘positive reinforcement’ (n = 188; 42.7% of responses), creating a ‘calm environment’ (n = 138; 31.4% of responses), ‘providing safety’ (n = 121; 27.5% of responses), ‘stopping exposure’ (n = 51; 11.6% of responses), and ‘using alternative therapies’ (n = 3; 0.7% of responses).

‘Going slowly’ included slow exposure to new exposures, providing a gradual increase and repetition of exposure, having patience, and slowly desensitising kittens to a new exposure (i.e. the desensitisation portion of a desensitisation and counterconditioning [D&CC] protocol). For example, P207 explained, *“I introduce the new exposure slowly and in a graded manner”* while P213 described, *“Slow, patient introductions on multiple occasions.”*

‘Positive reinforcement’ included providing treats, food, or encouragement (i.e. the counterconditioning portion of a D&CC protocol) to make the experience positive for the kitten. For instance, *“Offer treats or soft food while experiencing the new exposure”* (P307).

‘Calm environment’ included sitting quietly nearby, talking softly, or providing soft, ambient noise. Some participants made this part of their everyday activities, for example: *“Typically I spend time in the same room as the kitten. I will read a book, answer emails, write a letter‚ calm activities that show I’m not a threat while we share space”* (P335).

Relatedly, ‘safety’ focused on comfort for the kittens, providing hiding spots, and consolation. For example, P194 described, *“Making* [kittens] *feel as safe and comfortable as possible before, during, and after the exposure.*” Participants also mentioned that they would ‘stop exposure’, if needed, or decrease the level of exposure based on the kitten’s reaction. For example, P156 explained, *“If the* [kittens] *really seem overly afraid, I’d probably remove them from the situation calmly or stop exposure.”* A few participants used ‘alternative therapies’, including the use of anti-anxiety drops or Feliway® (P450). In summary, participants socialised fearful kittens by modifying their methods of interaction and making environmental changes to support kitten needs.

Participants also considered kitten traits when providing socialisation which included providing ‘agency’ (n = 124; 28.2% of responses), ‘handling’ (n = 123; 28.0% of responses), using ‘play’ (n = 92; 20.9% of responses) or a ‘social mentor’ (n = 48; 10.9% of responses) to engage, and ‘understanding behaviour’ (n = 28; 6.4% of responses).

Providing the kitten with ‘agency’ was the most common factor mentioned and included letting *“the kitten make the decision to approach”* (P76) or allowing the kitten to *“explore the new stimulus without force”* (P458). Participants also observed how kittens responded to ‘handling’, including petting, cuddling, or grooming, as well wrapping the kitten in a blanket, such as a ‘purrito’ as a means of affection and comfort. For example, *“Provide comfort via petting if they respond positively to that”* (P236); *“Everyday try to handle kitten more and more”* (P433); and *“Quiet touch, hand feeding, swaddling”* (P293).

Participants described the use of ‘play’ through offering different types of toys or training. P198 explained they *“Use wand toys that enable more distant play but move the toy close whenever possible”* while also using *“slow head pets during play and eating”* and P427 stated “*I have found clicker training to be helpful with shy kittens.”* Another technique that participants considered helpful was use of a ‘social mentor’ (e.g. other kittens, littermates, the mother cat, household cat) to provide social learning or act as a ‘behaviour mentor.’ For example: *“If there is a littermate who is not acting fearfully, I will try to pair them up* [in the carrier or in an activity] *so they learn from each other”* (P350), and *“*[I] *often use social friendly cats to teach them how to interact with people and give them more confidence”* (P209).

‘Understanding behaviour’ included identifying, monitoring, or observing kitten body language and behaviour for signs of fear or stress. P175 described, *“Watching* [the kittens’] *body language closely to see when they are overstimulated,”* while P326 elaborated, *“Only progressing to the next step/level when no fear is shown,”* and P103 described they *“Reintroduce* [fearful stimulus] *at another time when the kitten is relaxed.”* In the instances above, participants described their observations of kitten behaviour and how they adjusted socialisation practices to decrease fear and build confidence.

Finally, 21 participants (4.8% of responses) noted they had never fostered a fearful kitten and 45 participants (10.2% of responses) described practices that are not currently recommended for socialising kittens, such as ignoring the kitten, not providing hiding spots, limiting agency, forcing the interaction, or flooding the fearful kitten with the exposure (American Association of Feline Practitioners 2004; Seksel 2008). For example, P262 explained, *“Removing the fearful kitten from their littermates, forcing them to interact with the fearful exposure”* while P317 mentioned they *“Minimize hiding areas.”*

### Resources needed to conduct socialisation

Participants described a variety of resources which we categorised into three main themes: (1) shelter-supplied support; (2) personal knowledge and external support; and (3) having access to socialisation opportunities. Of the 487 responses, 402 participants answered this question.

‘Shelter-supplied support’ included communication (n = 199; 49.5% of responses), education (n = 149; 37.1% of responses), medical care (n = 71; 17.7% of responses), availability of help (n = 61; 15.2% of responses), supplies (n = 45; 11.2% of responses), behaviour support (n = 27; 6.7% of responses), and reinforcement (n = 16; 4.0% of responses).

The most mentioned factor was ‘communication’, which included any support from the shelter via telephone or email. For example, P32 described, *“I know that our fosters are always appreciative when they can talk to someone either on the phone or in person about issues they’re having socializing.”* Participants especially appreciated clear communication, as P65 wrote, *“It’s really helpful to have experts to ask questions. There’s tons of (often conflicting) kitten advice on the internet, so it’s important to me to have a reliable, accessible source.”* ‘Availability’ of shelter support outside of regular business hours (e.g. emergency or 24/7 support) was also important, as P27 appreciated, *“After hours contact for situations I haven’t encountered before or emergencies.*”

‘Education’ included shelter-supplied materials such as hand-outs, manuals, training workshops, or checklists of socialisation guidelines, recommended exposures, or protocols. As examples: *“I think providing a hand-out with summarized key points is useful, as well as ensuring foster families undergo an orientation training of some kind”* (P1); and *“I think a resource guide to how to introduce kittens to new sights, sounds, and stimul*[i] *in a way that’s safe and gentle for them would be incredible. Practical suggestions on ways to build confidence and let them express natural behaviors would be beneficial too”* (P198).

‘Medical care’ included any support for health or veterinary care or services, including through telemedicine, and availability of necessary medication. For example, P61 described, *“My shelter has great vet resources (they have a full-time vet) and it is very reassuring to know I can call and bring the kittens in within 24 hours if needed. That makes me feel very supported!”*

In addition, ‘supplies’ included items provided by the shelter, such as treats, food, toys, litter, neonate supplies, as well financial assistance for supplies was important. P155 described, *“I was given toys, food, and other kitten supplies”* and P33 added, *“We have a team for newborn/bottle feeding with kits you receive that have a scale, syringes, KMR* [kitten milk replacer]*, heating stone, blanket, booklets, etc.”*

‘Behaviour support’ included behaviour-specific support from the shelter or having access to a behaviour expert for advice, such as *“Having a behaviour team to ask advice from”* (P129) and *“Behavioural staff (trained in shelter behaviour) is available for any concerns regarding socialization. Working out a plan together for any kitten concerns is essential”* (P201).

Finally, ‘reinforcement’ included receiving emotional support or affirmation, and being told from the shelter that the foster caretaker is doing a good job. For example, P10 described, *“Complimenting when the kittens are well socialized when returning to the shelter”*, P366 added, *“Feeling like you are heard”* and P50 elaborated, *“Emotional support with tough cases. We’re a family.”* Thus, shelter-supplied support was diverse, ranging from the provision of veterinary and other supplies to foster caretakers feeling appreciated and part of a community.

‘Personal knowledge and external support’ included knowledge or support acquired from outside of the shelter, such as access to mentorship or a network of experienced foster caretakers (n = 112; 27.9% of responses), personal internet searching using Google or social media (e.g. Instagram, TikTok) (n = 24; 8.5% of responses), as well as independent foster knowledge and previous experience (n = 29; 7.0% of responses).

‘Mentorship’ was the most mentioned factor and included having a network of other foster caretakers, either in-person or online via Facebook, Slack, or other platform, and included receiving information, training, and mentoring from experienced foster caretakers. For example, P15 described, *“Facebook groups where you can bounce ideas off other fosters.”* Additionally, P151 valued *“Having an open communication channel for real time questions has been key to success — if we have a question about a kitten’s behavior or need a second opinion, it’s nice to have a group of experienced fosters and/or rescue volunteers to reach out to.”*

‘Online sources’ of information, such as Google or social media platforms (e.g. Instagram, Twitter/X, TikTok*)*, as well as well-known cat behaviour experts with online resources, such as Jackson Galaxy and Hannah Shaw, aka ‘Kitten Lady’, were also reported. ‘Kitten Lady’ (and/or her book, *Tiny but Mighty*) was mentioned frequently enough to warrant a separate code (‘Kitten Lady’: n = 29; 7.0% of responses). For example, P474 described how they *“learn/seek out most information on my own from my own vet and online resources and webinars”*, and P132 wrote *“I looked up all socialization techniques on YouTube and followed a few content creators on social media.”* As well, ‘foster knowledge’ included what foster caretakers learned from their own previous experiences or self-teaching. P308 explained, *“Usually the experienced ones like myself are the ones to give advice”* while P61 added, *“Most of what I know I’ve learned on my own through research.”* Thus, participants relied upon each other and online sources to learn about socialising kittens.

A final resource for conducting socialisation was ‘socialisation opportunities’ with humans (n = 6; 1.5% of responses) and animals (n = 3; 0.8% of responses). Regarding ‘human socialisation’, participants described the importance of *“visits from strangers”* (P2), a close foster-kitten bond (P142), as well as interactions with children (P191). ‘Animal socialisation’ included having access to animals, for example “*making sure kittens are with other kittens or mommas while young or other pets in the home”* (P336). Overall, foster caretakers appreciated having access to a variety of socialisation opportunities to provide social experiences.

Additionally, some participants noted a ‘lack of resources’ (n = 12; 3.0% of responses) from the shelter or about the fostering experience. For example, P61 wrote, *“I think educational resources are incredibly important but do not feel are provided by my current shelter.*” For some, communication speed was a problem: *“Currently response time from the shelter is very slow and this can cause unnecessary stress”* (P61). Finally, for others, human resources were lacking: *“Unfortunately what I have experienced is shelters and rescues are stretched so thin and/or rely mostly on volunteers there isn’t enough focus on support for fosters. After an initial volunteer training you’re pretty much on your own”* (P385). In contrast, *‘*none’ (n = 29; 7.2% of responses) was coded for participants’ descriptions of not needing support or not seeing socialisation resources as necessary to fostering. For example, P5 explained, *“I don’t think socialization help is necessary.”*

### Barriers to conducting socialisation

Participants described challenges and barriers they face while socialising kittens, which we categorised into three themes: (1) personal challenges; (2) shelter-specific challenges; and (3) kitten- or cat-related challenges. Of the 487 responses, 420 participants answered this question.

‘Personal challenges’ included participants’ descriptions of limitations due to time commitments outside of the home (n = 67; 16.0% of responses), household demographics (n = 45; 10.7% of responses), other pets in the home (n = 21; 5.0% of responses), space such as house layout or area (n = 16; 3.8% of responses), lack of experience (n = 9; 2.1% of responses), and energy including the emotional toll of fostering (n = 8; 1.9% of responses).

‘Time’ was the most mentioned factor and included mention of limitations due to time, having an uncertain schedule (P148), or full-time work or responsibilities outside of the home causing foster caretakers to be away from kittens for a period of time (P57, P252).

‘Household demographics’ included limitations due to household structure, and participants described how things such as *“lack of access to dogs and children”* (P49, P61) made it difficult to expose kittens to novel situations. For some, ‘other pets’ in the home raised concerns about behaviour conflicts and following safety protocols that required keeping household pets separate from foster kittens. P18 described, *“My cat doesn’t like other fosters so they need to stay separate,”* while P269 explained, *“Current dog is not cat-friendly so limits being able to introduce kittens to dogs.”* Finally, participants noted ‘space’ limitations due to the layout within the foster home, such as having a *“small space”* (P47), or *“not having stairs”* (P4), or *“no window access to expose* [kittens] *to more”* (P463).

Foster caretakers also perceived ‘lack of experience’ and ‘energy’ as barriers. Participants noted having insufficient knowledge and education about socialisation. ‘Energy’ included limitations due to the energy expenditure or emotional toll involved in fostering. P71 described, *“Now that I’m back working, it’s harder to carve out a lot of time and energy”* and P418 added that it is *“Emotionally hard at times to see limited progress.”* Overall, these personal challenges demonstrate how foster caretakers managed the daily work involved with caring for kittens in their homes.

‘Shelter-specific challenges’ included participants’ descriptions of lack of shelter support (n = 23; 5.5% of responses), and limitations due to cost (n = 10; 2.4% of responses) and access to medical care (n = 8; 1.9% of responses).

‘Lack of shelter support’ was the most mentioned factor and included limited contact with or support from the shelter, or limited shelter-provided supplies or educational guidance or training. P298 described, *“Sometimes shelters are limited on resources (toys, nutritious food, and supplies).”* P305 added, *“Lack of communication between myself and shelter personnel,”* and P212 expressed, *“I would love access to a ‘handbook’ of protocols/recommendations […] I would also love more guidance on the types of ways we should provide enrichment/socialization.”*

Specific limitations included ‘cost’ and limitations due to money or the financial responsibilities of fostering. For example, *“Money — if I had more expendable income, I could get the* [kittens] *nicer toys and resources”* (P62). Additionally, ‘access to medical care’ was a challenge. Participants described how *“Kittens get sick very often and it stops their socialization progress”* (P288) and that a *“Lack of timely veterinary services”* (P305) can limit socialisation advancement.

‘Kitten- or cat-related challenges’ included challenges related to the kittens themselves or the mother cat. This included illness (n = 34; 8.1% of responses), kitten behaviour (n = 16; 3.8% of responses), late intake into foster care (n = 9; 2.1% of responses), and challenges due to the mother cat (n = 7; 1.7% of responses).

‘Kitten illness’ included challenges related to kittens being sick, such as quarantine requirements while treating ringworm or due to not being vaccinated. For example, P65 described, *“It can be really difficult when the kittens are sick. You can’t gradually build up trust when you have to forcibly give medication.”* P234 noted this affected socialisation progress as well: *“If we have a single kitten that is sick and they can’t interact with other kittens this will be a challenge to socialize them properly.”*

‘Kitten behaviour’ included challenges due to kitten behaviour or personality, either individually or dynamics within a litter. P195 described this situation involving *“Uneven character traits within a litter — one very active, attention-seeking, aggressive kitten, one very reserved one,”* and, similar to kitten illness above, P144 explained how setbacks occur, *“At times the kittens need to be brought in for exams and/or surgery. If they are feral or shy and just making progress, the trip to a vet can sometimes set them back.*” Some participants understood behavioural links to ‘late intake’, that is, delayed arrival into foster care or into the shelter system, being removed late from a community colony, or not having prior experience with people due to late intake into foster care. For example, *“The kittens we work with are from outside so they have minimal to no positive interactions with humans”* (P52) and *“Lack of resources to trap the kittens at a younger and more impressionable age”* (P188).

As well, ‘mother cat’ behaviour could be challenging, and participants described how this impacted kitten socialisation. For instance, *“If mum is aggressive and ultra-protective of her kittens”* (P147) and *“Trying to navigate socializing the kittens without upsetting their mom”* (P31). Each of the sub-themes above indicate the difficulty of navigating socialising kittens when encountering health, behavioural, and other challenges.

### Quantitative results

The main quantitative outcome of interest was whether or not foster caretakers mentioned the use of a technique that is not recommended when providing socialisation to a fearful kitten based on current expert recommendations (e.g. American Association of Feline Practitioners 2004; Seksel 2008). Using a multivariable logistic regression model, we found that foster caretakers with a higher score for the personality trait of agreeableness had significantly lower odds of mentioning a non-recommended socialisation technique (Table 2). We also found that participants whose fostering practices were affected because of the COVID-19 pandemic had significantly greater odds of mentioning a non-recommended socialisation technique compared to those who reported no changes and to those who reported only fostering during the pandemic (Table 2). There were no issues with collinearity or violations of the linearity assumption, so no independent variables were removed, transformed, or categorised in the final model.

**Table 2.** Results from a multivariable logistic regression model investigating factors associated with whether or not kitten foster caretakers (n = 440) mentioned the use of a non-recommended technique when providing socialisation to a kitten reacting fearfully to a new exposure

Variable	OR	*P*-value	95%CI
Agreeableness personality trait	0.69	**0.021**	0.50, 0.95
Pandemic impacts^a^^,^^b^			
I have only fostered during the pandemic vs No, there have been no changes	0.66	0.530	0.18, 2.41
Yes, the pandemic impacted fostering vs No, there have been no changes	2.74	**0.005**	1.35, 5.55
Yes, the pandemic impacted fostering vs I have only fostered during pandemic	4.15	**0.024**	1.20, 14.32

a Qualitative question: *“We recognize that the COVID-19 pandemic has changed many people’s lifestyles and routines, and this may have impacted how we interact with foster kittens. Have your fostering practices changed because of the pandemic?”* with an open text box for “Yes” responses. See b.

b Open text responses included: “limited socialisation exposures,” “no in-person meet and greets or adoption events; virtual instead,” “reduced support,” “increased costs,” “stopped fostering,” “more time at home,” “able to foster more kittens.”

#### Pandemic-related qualitative descriptions

Finally, the pandemic question included in the quantitative model gave foster caretaker participants the opportunity to describe the impact of the pandemic on their socialisation practices. Responses included having limited access to socialisation exposures, such as “*Less variety of people for the kittens to interact with during socialization”* (P15) and *“Less socialization with friends and family. Litters were only exposed to people in our household during foster time”* (P31). Additionally, participants described meet-and-greets or adoption events were no longer in-person, they had reduced support from the shelter, increased costs, or that they had stopped fostering altogether. In contrast, other foster caretakers mentioned that the pandemic allowed them to foster more kittens or spend more time at home due to no longer working in office or outside of the home because of public health restrictions. For example, “*I’ve had time to foster more kittens; however, training and mentoring other fosters has become more challenging”* (P56).

## Discussion

This study highlights current experiences and practices around kitten socialisation by volunteer foster caretakers, with a focus on the types and timing of social and non-social opportunities provided to kittens, kitten behaviours or characteristics perceived as important for successful adoption, and risk factors for use of non-recommended socialisation techniques when working with fearful kittens. The mixed-method survey approach allowed for a rich investigation into foster caretaker experiences with socialisation practices, important resources, and key challenges, and can be used to guide educational and support resources.

Kittens are receiving a wide variety of relevant exposures during their socialisation period, such as general handling, different types of toys, hiding areas, and designated scratching materials. However, key exposures (e.g. handling that mimics veterinary examinations and meeting unfamiliar animals) were lacking. Appropriate early exposure is important for behavioural development, to reduce the likelihood of future behavioural issues, such as aggression, and to help kittens receive more thorough veterinary care with less stress (Levine 2008). Additionally, we found that a high proportion of foster caretakers used many positive and evidence-based approaches to providing socialisation to kittens who are responding fearfully to new exposures, such as providing the kitten with agency and choice to engage with the exposure, and providing slow, calm, and safe environments with positive reinforcement (Seksel 2008; American Veterinary Medical Association 2015; Howell *et al.* 2015). These approaches are important to ensure kittens develop positive early associations to the world around them.

A number of participants in the current study mentioned the use of techniques that are not recommended when socialising fearful kittens (e.g. American Association of Feline Practitioners 2004; Seksel 2008), and this was unexpected given these methods are generally discouraged by animal welfare organisations (e.g. BC SCPA undated). Denying kittens (or any animal) agency to engage with their environment can negatively impact animal welfare (Špinka & Wemelsfelder 2018; Špinka 2019), and preventing a fearful kitten from retreating from a fear-provoking stimulus or conducting exposure at full intensity can cause overstimulation and flooding, which can lead to an increased and prolonged fear response or other behavioural issues such as aggression (Yin 2009; Landsberg *et al.* 2013; Riemer *et al.* 2021).

The foster caretaker personality trait of agreeableness was protective against mentioning the use of a non-recommended socialisation technique. A person with a high level of agreeableness is generally warm, friendly, and considerate, with an optimistic view of human nature, gets along well with others, and is seen as compassionate, affectionate, and trustful (McCrae & Costa 1999) and the trait has been linked to cognitive and affective empathy (Melchers *et al.* 2016). Human personality traits have been found to influence the relationships people have with cats as well as cat behaviour in the home (Wedl *et al.* 2011; Finka *et al.* 2019; Finka 2022). For example, higher scores of agreeableness in cat caretakers are associated with a higher level of caretaker-reported satisfaction with their cat (Finka *et al.* 2019). In other species, dog handlers with a higher score for agreeableness were less likely to use verbal correction when training their dogs (Payne *et al.* 2015). In humans, higher maternal agreeableness is negatively associated with the use of a harsh or coercive parenting style among parents of dysregulated children, suggesting more agreeable parents promote positive emotional regulation in their children (Coplan *et al.* 2009). The agreeableness personality trait in feline caretakers may confer inherent advantages as regards appropriately socialising kittens and may benefit fearful kittens. It is also possible that people with higher agreeableness may be more willing to foster fearful kittens or even foster kittens in general, reflecting a potential bias in the current study population. Data obtained from online surveys may also be prone to selection bias (Gosling *et al.* 2004) and this may have occurred with our study population. Further research is needed to explore whether agreeable foster caretakers are less likely to use negative socialisation techniques due to their compassionate nature, what challenges might lead to even an agreeable person resorting to non-recommended techniques, and whether human personality screening is beneficial for shelters seeking foster caretakers, particularly for fearful kittens. Additionally, future investigation into the impact of human personality on our relationships with kittens, as well as the use of more in-depth personality assessments (e.g. the 44-item Big Five Inventory; John & Srivastava 1999), is important for understanding the impact of human personality on kitten and cat behaviour.

The second factor that was associated with reported use of a non-recommended technique for socialising a fearful kitten was the impact of the COVID-19 pandemic on fostering practices. It has been found that shelter support was particularly important for foster caretakers over the pandemic (Reese *et al.* 2022): given increased staffing pressures, shelters may have reduced their support to foster caretakers during this time. Compared to participants whose fostering practices were not impacted by the pandemic and to those who had only fostered kittens during the pandemic, foster caretakers whose practices were altered because of the pandemic had greater odds of mentioning the use of a non-recommended technique. The onset of the pandemic dramatically changed the world as we knew it, including our care of companion animals. Companion animals provided substantial support for their caretakers to mitigate the emotional effects of lockdowns (e.g. Bowen *et al.* 2020; Martin *et al.* 2021) and fostering appeared to increase primarily because people were spending more time at home (Reese *et al.* 2022); however, availability of veterinary care was a major concern because of pandemic restrictions (Owczarczak-Garstecka *et al.* 2022) and access to socialisation opportunities for young animals was also impacted during periods of physical distancing. It is not surprising that the practices of foster caretakers in the current study were altered because of the pandemic; however, being more likely to use a non-recommended technique for socialising a fearful kitten because of the pandemic was an interesting finding. There is the chance of increased frustration, anxiety, or pessimism due to the pandemic among the population in general (e.g. Vitorino *et al.* 2022). The non-recommended techniques used for socialising fearful kittens that were mentioned in this study can likely be eased with improved or standardised training. However, further research is needed to understand the specific impacts of the pandemic.

There may have been issues for some participants with interpreting what represents a ‘fearful kitten.’ When asked about their approach to providing socialisation to a kitten reacting fearfully to a new exposure, some participants indicated they had not fostered any fearful kittens or that they had not fostered ‘feral’ kittens, implying potential misinterpretation of what ‘fearful’ looks like. Though kittens and cats are known to show individual differences in behaviour (e.g. Feaver *et al.* 1986; Stella & Croney 2019), it is unlikely that a kitten had *never* displayed any level of fear toward new environments or new exposures as this is a common response for cats encountering a novel situation (Rodan 2010). This disconnect between linking external behaviour and internal emotional state could hinder appropriate responses to a kitten displaying subtle or overt signs of fear and may cause preventable damage to long-term welfare.

We have previously found that mild fear is more challenging to interpret in young kittens compared to neutral or positive states (i.e. no fear) or moderate fear states, but also that specialised training in identifying kitten behaviour is effective at improving human interpretation of kitten emotional states (Graham *et al.* 2024a). Foster and kitten caretakers should also consider differences within individual kitten responses to different stimuli, recognising when kittens are fearful, and adapting socialisation practices as needed (Graham *et al.* 2024b). Understanding kitten behaviour was mentioned by participants as a factor used for socialising a fearful kitten. Additionally, having access to behaviour-specific support was mentioned as a key shelter-supplied resource, and issues regarding kitten behaviour were mentioned as a kitten-related challenge. Therefore, tailored educational interventions in identifying subtle kitten behaviour and making the connection to emotional state may aid foster caretakers’ interpretation. These findings suggest that understanding of and having support for kitten behaviour are important components for providing optimal socialisation within this population of foster caretakers.

Greater focus was given to the later weeks of the socialisation period for kittens (i.e. primarily from 4 to 9 weeks of age; Figure 2), with fewer foster caretaker participants focusing on socialisation during the early weeks (i.e. weeks 2 and 3). While the later weeks of a kitten’s socialisation period are likely the most important, as kittens are more mobile and have increased social and environmental interactions (Bateson 2014), this finding highlights the need to improve education on the importance of starting socialisation early in a kitten’s life. Kittens may not be rescued to a shelter or placed in foster homes as early as two weeks of age, but this finding acts as a reminder to ensure fostering organisations are emphasising the full range of this important window in a kitten’s development (Karsh 1984; Karsh & Turner 1988; Lowe & Bradshaw 2001; Bateson 2014).

Participants reported that kittens who are aggressive and fearful of people and other animals may be less likely to be adopted permanently. Yet, when examining the socialisation opportunities and exposures provided to kittens within foster care, less than a third of foster caretakers (26.3%) exposed their kittens to unfamiliar animals. This demonstrates a disconnect between expectation and reality, which can hinder the success and longevity of keeping kittens in their adoptive home. Many participants mentioned that they do not have access to other animals during their kittens’ socialisation period, which highlights the need for shelters to facilitate access to these important opportunities. This finding also highlights the importance of creating positive early life experiences and understanding caretaker expectations for beneficial outcomes later in life in an effort to retain kittens in the home.

Other prominent resources mentioned by participants as necessary to conduct the best socialisation for their kittens included clear and timely communication with emotional and financial support, education in the form of hand-outs or checklists, and access to a mentorship network with knowledgeable and experienced peers. Support for foster caretakers became increasingly important during the pandemic, particularly in terms of access to a mentorship network, being provided with emotional support, maintaining high levels of communication, and receiving monetary support for the care provided (Reese *et al.* 2022). These concerns are echoed in the current study as important resources and challenges faced by kitten foster caretakers. It is also important for shelters to be aware of these needs to better support their foster caretakers in performing the best care and socialisation possible.

Limitations of this study include some forms of potential bias. We potentially encountered sampling bias due to the self-selection of participants, where we may have an over-representation of foster caretakers interested in or motivated towards good welfare for kittens. These individuals might be more likely to conduct positive socialisation practices and may hold more awareness of the socialisation period and optimal practices. In addition, the vast majority of participants identified as women, which has been observed in previous studies of human-companion animal relationships (e.g. Owczarczak-Garstecka *et al.* 2022). Future research should try to recruit participants representing other gender identities to minimise this bias and allow generalisability and investigation of gender effects. Finally, we attempted to mitigate recall bias by limiting our recruitment to those who had fostered kittens within the past two years from the study participation date; however, issues with recalling earlier experiences may still have been present. Additionally, while we did receive responses from foster caretakers around the world, the majority of responses were from those located in the USA or Canada and, therefore, the findings may not fully reflect the experiences and practices in other regions.

Overall, foster caretakers provide kittens with a number of beneficial socialisation opportunities, with room for improvement around exposure to mimicking handling during a vet visit and unfamiliar animals, with starting socialisation as early as possible, and with adapting socialisation practices for fearful kittens. Participants highlighted key resources they require from shelters for effective socialisation, such as having access to appropriate educational tools, clear and timely communication, and ample support. Further, human personality characteristics were associated with socialisation practices that can impact kitten behavioural development and further research is needed to broaden understanding of this relationship. Future research can also investigate the guidelines provided to foster caretakers and where there are opportunities for improvement. In our survey we included questions about shelter guidelines being provided to foster caretakers (Table S1; Supplementary material). In future analyses, these questions will be investigated both quantitatively and qualitatively in a similar approach to the current study, with the ultimate goal of improving kitten welfare and behavioural development for long-term adoption and caretaker satisfaction.

### Animal welfare implications

Foster caretakers hold essential first-hand perspectives of the early weeks of a kitten’s life. This study described the experiences foster caretakers provide to socialise kittens, and highlighted room for improvement as regards the process of socialising fearful kittens in particular. Improving our understanding of how early socialisation is provided to kittens, the resources foster caretakers need, and the challenges they face will allow for catering guidelines to improve behavioural development and long-term kitten welfare and help to retain kittens in their adoptive homes. This study also highlighted the impacts of the COVID-19 pandemic on fostering practices, which also has implications for kitten welfare. Understanding how best to support the humans caring for young kittens will ultimately improve our ability to provide optimal socialisation experiences during this critical period in a kitten’s life.

## Supporting information

Graham et al. supplementary material Graham et al. supplementary material

## Supplementary material

The supplementary material for this article can be found at http://doi.org/10.1017/awf.2024.45.
