# Supplementary material for: Understanding kitten fostering and socialisation practices using mixed methods
Source: Anim Welf. 2024 Nov 11;33:e52. doi: 10.1017/awf.2024.45 (PMC11655271; doi:10.1017/awf.2024.45)
Supplement: Graham et al. supplementary material [file S0962728624000459sup001.pdf]

Table S1. Overview of a mixed methods survey completed by kitten foster parents titled *Kitten fostering experiences and practices.*

START AND CONSENT

INCLUSION QUESTIONS

If you have read the above information and agree to participate in this study, please select "Yes".

Yes

No

(If No is selected, Skip to End of Survey)

(Mandatory to answer)

Are you at least 18 years of age?

Yes

No

(If No is selected, Skip to End of Survey)

(Mandatory to answer)

Have you fostered kittens (8 weeks of age or younger) <u>within the last 2 years</u>?

Yes

No

(If No is selected, Skip to End of Survey)

(Mandatory to answer)

We recognize that the COVID-19 pandemic has changed many people's lifestyles and routines, and this may have impacted how we interact with foster kittens as well. Please answer the following questions considering the **normal** (i.e., pre-pandemic) state, behaviour, and management of your foster kittens. At the end of the survey, we will ask you about how these practices may be different during the pandemic or if you have only fostered kittens during the pandemic.

Please note there is no word limit for open-ended questions.

Previous experience

How many years have you been fostering kittens?

Less than 1 year, 1, 2, 3, 4…20+ *(dropdown list)*

Approximately how many <u>litters</u> of kittens have you fostered <u>in total</u> (over your entire life)? This can include groups of kittens from multiple mothers.

1, 2, 3, 4…20+ *(dropdown list)*

On average, how many <u>litters</u> of kittens do you foster <u>per year</u>? This can include groups of kittens from multiple mothers.

1, 2, 3, 4…20+ *(dropdown list)*

How many different shelters or rescues have you ever fostered for?

1, 2, 3, 4…20+ *(dropdown list)*

Briefly, why do you foster kittens?
(Text box for open-ended response)

Have you ever held any of the following positions related to companion animal health and behaviour? Please select all that apply.
None
Cat or Dog Trainer or Behaviour Consultant
Veterinarian
Veterinary Technician, Assistant, Nurse, or Student (or other position within a veterinary clinic setting)
Shelter Worker or Volunteer (or other position within a shelter setting)
Groomer
Animal Behaviour/Welfare Researcher or Student
Other, please describe______________

Please answer the following questions thinking about the kittens you have fostered over the past 2 years who were 8 weeks of age and younger. Please note there is no word limit for the open-ended questions.

Aside from foster kittens, have you had other animals living in your home during this period? Please select all that apply.
No other pets
Cats
Dogs
Rabbits
Small mammals (e.g., hamsters, guinea pigs, mice, rats)
Reptiles/amphibians (e.g., frogs, lizards, turtles, snakes)
Birds
Fish
Other, please describe______________
(If "No other pets" is selected, skip next question)

On average, how have you allowed your foster kittens interact with other animals in the home? Please select all that apply.
Free interacting or play (i.e., unsupervised)
Controlled, supervised interacting or play only
Feeding in the same room
Visual access only (i.e., separated by a barrier but still within visible range)
They did not interact
Other, please describe______________

Socialization practices

The following questions are about the socialization practices and techniques you typically use with your foster kittens and how your kittens respond. Socialization includes exposing your kittens to new people, environments, things, and situations and how this exposure is conducted. Please answer the questions about your kittens' experiences and behaviours during their first 8 weeks of age (e.g., if the kittens are still in your care when they are 10 weeks old, please only answer about them when they were 8 weeks of age and younger).

On average, how much time in a day have you or other people in your household spent actively playing or interacting with your foster kittens?
0 to 1 hour
1 to 2 hours
2 to 3 hours
3+ hours

Did you give names to your foster kittens?
Yes
No

Did your foster kittens get access to any of the following while they were in your care? Please select all that apply.

	Yes	No	N/A
**Small toys** (e.g., balls with bells, furry mice, crinkle balls, stuffed toys)	○	○	○
**Interactive toys** (e.g., feather wands, string toys)	○	○	○
**Explorative items** (e.g., boxes, tunnels, paper bags)	○	○	○
**Puzzle feeding devices** (e.g., puzzle feeders, food hiding toys)	○	○	○
**Elevated platforms** (e.g., perches, cat tree, furniture)	○	○	○
**Designated scratching materials** (e.g., scratching post, horizontal scratching mat, designated floor/carpet areas)	○	○	○
**Hiding areas** (e.g., covered beds, boxes, underneath furniture)	○	○	○
**Scent stimulation** (e.g., catnip, silver vine)	○	○	○
**Visual stimulation** (e.g., view of a window, aquarium, TV/tablet screen)	○	○	○
**Training** (e.g., to do tricks or respond to commands)	○	○	○
**Supervised outdoor access** (e.g., fenced backyard, patio, catio, on leash)	○	○	○
**Unsupervised outdoor access** (i.e., free roaming)	○	○	○
Other, please describe_____________	○	○	○

Did you expose your foster kittens to any of the following while they were in your care? Please select all that apply.

	Yes	No	N/A
**Transportation** (e.g., cat carrier, car rides)	○	○	○
**Noisy household objects** (e.g., vacuum, broom, plastic bags, blow dryer, pots clanging, blender)	○	○	○
**Movement opportunities** (e.g., climbing over an obstacle, climbing stairs)	○	○	○
**Different surfaces** (e.g., wood, metal, carpet, elevated, wobbly)	○	○	○
**Auditory stimuli** (e.g., doorbell, phone ringing, alarm ringing, children playing/screaming, music, sirens, construction, fireworks)	○	○	○
**Handling** (e.g., picking up, petting, grooming, clipping claws)	○	○	○
**Mimic vet visit** (e.g., gently opening eyes/pressing nose/opening mouth; hold and "examine" body, head, paws, limbs; gently pinch skin to mimic vaccine; wipe and cover with towel; hold on countertop/raised surface)	○	○	○
**Unfamiliar people** (e.g., members of other genders, different facial hair, people with hats/jackets, children, different ethnicities)	○	○	○
**Unfamiliar animals** (e.g., friend's pet coming into the home, neighbour pet)	○	○	○
Other, please describe____________	○	○	○

If you had the kittens' mother with you in foster care, did you alter how you provided any of the above exposures or socialization practices?
I have never had the kittens' mother with me in foster care
No, I did not do anything different
Yes, please describe____________

Please describe your approach to providing socialization to a kitten who is reacting fearfully to a new exposure.
(Text box for open-ended response)

On average, which weeks of age do you focus on for providing your kittens with socialization? Please select all weeks that apply.
Week of age

0	1	2	3	4	5	6	7	8	9	10	11	12	13	14	15	16
○	○	○	○	○	○	○	○	○	○	○	○	○	○	○	○	○

At what approximate age are your foster kittens typically available for adoption?
Younger than 6 weeks, 6 weeks, 7 weeks, 8 weeks, …20 weeks+ (dropdown list)

Does the shelter(s) you foster for provide guidelines or recommendations for kitten fostering and care?
Yes
No
I don't know
(If Yes is selected, prompt the next 5 questions)
Do the shelter guidelines or recommendations include information specific to socializing your foster kittens (i.e., exposing your kittens to new people, environments, things, and situations and how this exposure is conducted)?

Yes
No
I don't know

Please describe aspects of the shelter's guidelines or recommendations you feel are the most important for successful and long-term adoption (i.e., keeping kittens in their adoptive home with high adopter satisfaction).
(Text box for open-ended response)

Why do you feel these guidelines or recommendations are the most important?
(Text box for open-ended response)

Please describe aspects of the shelter's guidelines or recommendations you feel may reduce success of long-term adoption (i.e., could make kittens less likely to stay in their adoptive home, and could reduce adopter satisfaction).
(Text box for open-ended response)

Why do you feel these guidelines or recommendations may reduce the success of adoption?
(Text box for open-ended response)

When thinking about successful and long-term adoption in general (i.e., keeping kittens in their adoptive home with high adopter satisfaction), please rate the following characteristics in terms of enhancing or reducing the success of adoption.

Enhancing = the characteristic helps kittens get adopted, makes it easier for kittens to stay in their adoptive home, and can enhance adopter satisfaction

Reducing = the characteristic reduces kittens' chances of getting adopted, makes it more difficult for kittens to stay in their adoptive home, and can reduce adopter satisfaction.

**CHARACTERISTIC**	**Very reducing**	**Somewhat reducing**	**Neither reducing nor enhancing**	**Somewhat enhancing**	**Very enhancing**
**Active/energetic**	○	○	○	○	○
**Bold**	○	○	○	○	○
**Playful**	○	○	○	○	○
**Affectionate**	○	○	○	○	○
**Impulsive**	○	○	○	○	○
**Reactive/sensitive**	○	○	○	○	○
**Aloof/reserved**	○	○	○	○	○
**Submissive**	○	○	○	○	○
**Aggressive**	○	○	○	○	○
**Attention-seeking**	○	○	○	○	○
**Intelligent**	○	○	○	○	○
**Sociable with other animals**	○	○	○	○	○
**Sociable with people**	○	○	○	○	○
**Fearful of other animals**	○	○	○	○	○
**Fearful of people**	○	○	○	○	○
**Fearful of new objects or environments**	○	○	○	○	○
**Protective of people/caretakers**	○	○	○	○	○
**Protective of objects/food**	○	○	○	○	○
**Other, please describe______________**	○	○	○	○	○

Please describe the types of assistance or resources (e.g., educational resources, phone support) you feel are essential for you to feel supported by your shelter to conduct the best socialization for your kittens.
(Text box for open-ended response)

Please describe any challenges or barriers you feel reduce your ability to conduct the best socialization for your foster kittens. If no challenges or barriers, please say None.
(Text box for open-ended response)

Do you have anything else you would like to add about your kitten fostering experience, the socialization period, best practices for foster kittens, and/or kitten characteristics you feel are important for adoptability?
No
Yes, please describe__________

Demographics
What is your age (in years)?
18, 19, 20 …. 100+ (dropdown list)

What is your gender?
Man
Woman
Non-binary
Prefer to self-describe___________________
Prefer not to answer

What country do you live in?
All countries available (dropdown list)

What type of community do you live in?
Rural community (fewer than 4,999 people)
Small community (5,000 to 49,999 people)
Mid-sized community (50,000 to 999,999)
Large community (more than 1 million people)

How many adults (18+ years of age) live in your household (including yourself)?
1–10+ (dropdown list)

How many children and/or teenagers (under 18 years of age) live in your household?
0–10+ (dropdown list)

Ten-item personality inventory (TIPI)
Here are a number of personality traits that may or may not apply to you. Please select the rating next to each statement to indicate the extent to which you agree or disagree with that statement. You should rate the extent to which the pair of traits applies to you, even if one characteristic applies more strongly than the other.

Please select your agreement with each pair of traits.

PERSONALITY TRAITS	Disagree strongly	Disagree moderately	Disagree a little	Neither agree nor disagree	Agree a little	Agree moderately	Agree strongly
**Extraverted, enthusiastic**	O	O	O	O	O	O	O
**Critical, quarrelsome**	O	O	O	O	O	O	O
**Dependable, self-disciplined**	O	O	O	O	O	O	O
**Anxious, easily upset**	O	O	O	O	O	O	O
**Open to new experiences, complex**	O	O	O	O	O	O	O
**Reserved, quiet**	O	O	O	O	O	O	O
**Sympathetic, warm**	O	O	O	O	O	O	O
**Disorganized, careless**	O	O	O	O	O	O	O
**Calm, emotionally stable**	O	O	O	O	O	O	O
**Conventional, uncreative**	O	O	O	O	O	O	O

We recognize that the COVID-19 pandemic has changed many people's lifestyles and routines, and this may have impacted how we interact with foster kittens. Have your fostering practices changed because of the pandemic?
I have only fostered during the pandemic
No, there have been no changes
Yes, please describe__________ *(Text box for open-ended response)*

END

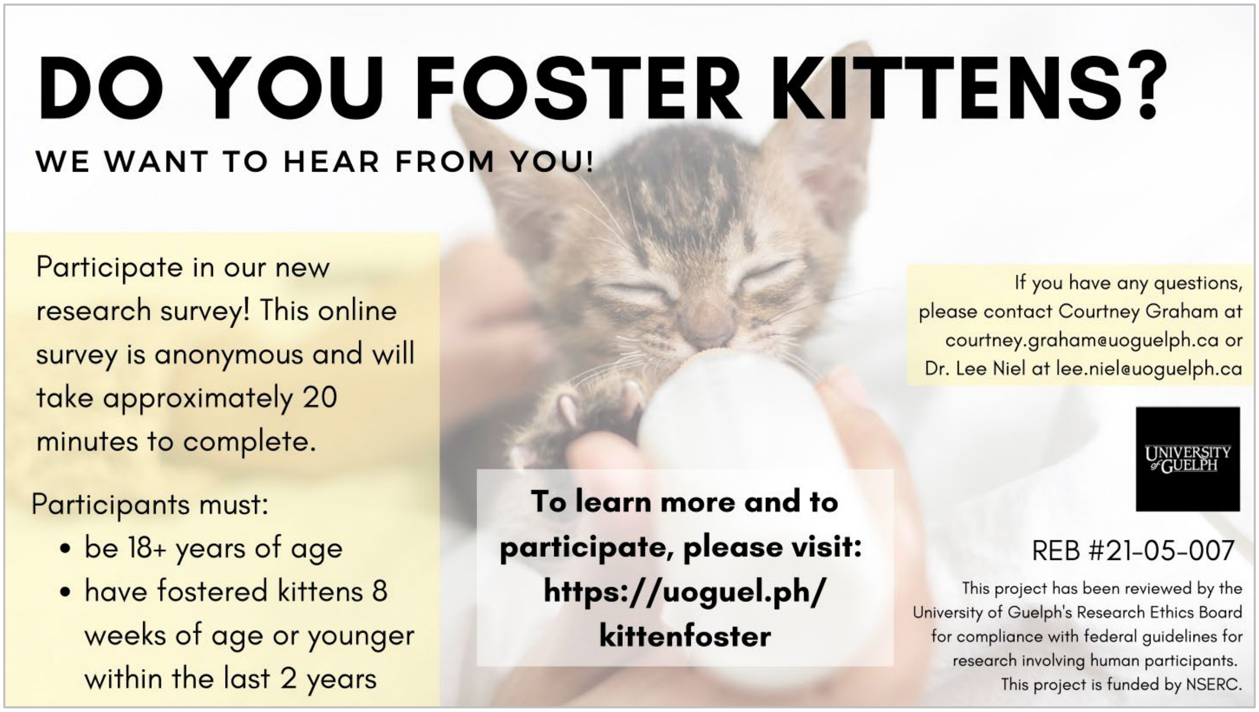

Figure S1. Advertisement for participant recruitment for the online survey titled *Kitten fostering experiences and practices.*
